# Amnio acid substitution at position 298 of human glucose-6 phosphatase-α significantly impacts its stability in mammalian cells

**DOI:** 10.1007/s00726-023-03263-8

**Published:** 2023-03-21

**Authors:** Jingsong Cao, Arianna Markel, Erin Hanahoe, Tatiana Ketova, Cosmin Mihai, Zach Zalinger, David Marquardt, Nicholas J. Amato, Yi Min Cheng, David W. Reid, Athanasios Dousis, Paloma H. Giangrande, Joshua R. Schultz, Paolo G. V. Martini, Patrick F. Finn

**Affiliations:** 1 Rare Diseases, Moderna, Inc., 200 Technology Square, Cambridge, MA 02139 USA; 2 Platform, Moderna, Inc., 200 Technology Square, Cambridge, MA 02139 USA; 3 Current Address: Tessera Therapeutics, Somerville, MA USA; 4 Current Address: Wave Life Sciences, Cambridge, MA USA

**Keywords:** Glycogen storage disease, Glucose metabolism, Protein folding, Degradation, Glycosylation

## Abstract

Glucose-6-phosphatase-α (G6Pase-α) catalyzes the hydrolysis of glucose-6-phosphate to glucose and functions as a key regulator in maintaining blood glucose homeostasis. Deficiency in G6Pase-α causes glycogen storage disease 1a (GSD1a), an inherited disorder characterized by life-threatening hypoglycemia and other long-term complications. We have developed a potential mRNA-based therapy for GSD1a and demonstrated that a human G6Pase-α (hG6Pase-α) variant harboring a single serine (S) to cysteine (C) substitution at the amino acid site 298 (S298C) had > twofold increase in protein expression, resulting in improved in vivo efficacy. Here, we sought to investigate the mechanisms contributing to the increased expression of the S298C variant. Mutagenesis of hG6Pase-α identified distinct protein variants at the 298 amino acid position with substantial reduction in protein expression in cultured cells. Kinetic analysis of expression and subcellular localization in mammalian cells, combined with cell-free in vitro translation assays, revealed that altered protein expression stemmed from differences in cellular protein stability rather than biosynthetic rates. Site-specific mutagenesis studies targeting other cysteines of the hG6Pase-α S298C variant suggest the observed improvements in stability are not due to additional disulfide bond formation. The glycosylation at Asparagine (N)-96 is critical in maintaining enzymatic activity and mutations at position 298 mainly affected glycosylated forms of hG6Pase-α. Finally, proteasome inhibition by lactacystin improved expression levels of unstable hG6Pase-α variants. Taken together, these data uncover a critical role for a single amino acid substitution impacting the stability of G6Pase-α and provide insights into the molecular genetics of GSD1a and protein engineering for therapeutic development.

**Supplementary Information:**

The online version contains supplementary material available at 10.1007/s00726-023-03263-8.

## Introduction

Glucose-6-phosphatase (G6Pase), which catalyzes the hydrolysis of glucose-6 phosphate (G6P) to glucose and inorganic phosphate, is a key enzyme operating in the last step of both glycogenolysis and gluconeogenesis and plays an essential role in maintaining glucose homeostasis in mammals (Foster and Nordlie 2002; Schaftingen and Gerin 2002; Hutton and O’Brien 2009). Three types of mammalian G6Pase homologues, encoded by three distinct genes (*G6PC* or *G6PC1*, *G6PC2*, and *G6PC3*) with unique tissue distribution patterns, have been identified so far (Lei et al. 1993; Arden et al. 1999; Martin et al. 2002). Among them, *G6PC* (encoding glucose-6-phosphatase-alpha, G6Pase-α) is predominantly expressed in liver and kidney where it functions as a key regulator in maintaining euglycemia in the fasted state (Hutton and O’Brien 2009; Chou et al. 2010). Mutations in the *G6PC* gene cause glycogen storage disease type1a (GSD1a) (Online Mendelian Inheritance in Man, OMIM # 232200), which is an autosomal recessive disorder with an incidence of 1 in 125,000 (Chou et al 2015). GSD1a is characterized by severe fasting hypoglycemia due to a failure in generating endogenous glucose from G6P. As a result, deficiency of G6Pase-α leads to accumulation of G6P, an intermediate metabolite of multiple metabolic pathways, causing abnormal accumulation of macromolecules including glycogen and triglycerides in affected organs such as the liver and kidneys and subsequent hepatomegaly, nephromegaly, and metabolic imbalances in circulation presented as lactic acidemia, hypertriglyceridemia, hyperuricemia, hypercholesterolemia (Chou et al. 2010, 2015). The long-term complications of GSD1a include hepatocellular adenomas and carcinomas (HCA/HCC) and end-stage kidney disease (Chou et al. 2010, 2015).

The current standard-of-care for GSD1a relies on strict dietary management aimed at maintaining euglycemia. This is achieved by frequent feeding of uncooked or modified cornstarch (for adults or older children) or gastric drip feeding of glucose (for younger children or infants) (Greene et al. 1976; Correia et al. 2008; Shah and O’Dell 2013). However, the effectiveness of dietary management on long-term metabolic complications including hepatocellular adenomas and carcinomas (HCAs/HCCs) remains unproven and its long-term therapeutic window has been reported to be narrow (Franco et al. 2005; Wang et al. 2011; Calderaro et al. 2012; Derks et al. 2017; Steunenberg et al. 2018).

Currently, gene therapies that use viral vectors are being pursued as additional treatment options for GSD1a patients and have shown promise in correcting hypoglycemia and preventing other metabolic abnormalities in GSD1a animal models (Zingone et al. 2000; Clar et al. 2015; Lee et al. 2015; Kim et al. 2017) and in a clinical trial (Weinstein 2020) (NCT03517085). However, the clinical application of these approaches remains limited due to the gradual loss of transgene expression over time, the potential risk of genotoxicity, and pre-existing neutralizing antibodies (Hareendran et al. 2013). Enzyme replacement therapy (ERT) has proven successful in treating inherited metabolic diseases, such as lysosomal storage diseases (Parenti et al. 2015). However, GSD1a ERT is challenging, mainly because the affected G6Pase-α is a multi-transmembrane protein localized deep in the endoplasmic reticulum (ER) membrane, prohibiting delivery of exogenously produced functional protein to deficient cells.

We and others have used mRNA-based technology as a platform to deliver proteins in vivo to restore activity and overcome deficiencies in inherited metabolic diseases. This novel platform utilizes endogenous intracellular machinery for the production and localization of therapeutic proteins, which circumvents the challenges of delivering transmembrane proteins by traditional ERT. The potential of mRNA-based therapies has been demonstrated in preclinical animal models for many liver metabolic diseases, including those that are caused by deficiency of transmembrane proteins localized in the ER, mitochondria, and lysosomes, or on the cell surface (An et al. 2017; Jiang et al. 2018; Roseman et al. 2018; Trepotec et al. 2018; Berraondo et al. 2019; Cao et al. 2019; DeRosa et al. 2019; Martini and Guey 2019; Truong et al. 2019; Zhu et al. 2019). Recently, we and others have also showed that delivery of G6Pase-α encoding mRNA via lipid nanoparticles not only restored euglycemia and alleviated GSD1a-associated metabolic abnormalities such as hepatic glycogen accumulation, but also prevented liver tumor formation in a mouse model of GSD1a (Roseman et al. 2018; Cao et al. 2021). Based on the favorable efficacy and safety profiles in preclinical studies, this engineered mRNA for GSD1a (mRNA-3745) is being evaluated in a clinical trial (NCT05095727).

To improve the potency of mRNA-based therapies for GSD1a, we have employed a computer-aided protein engineering approach to enhance translation, stability, and activity. To this end, we have conducted a sequence alignment analysis using > 100 mammalian orthologues of G6Pase-α, with the hypothesis that consensus (i.e., most frequently used) residues in a family of G6Pases may be favorable for stability and/or function. This led to identification of consensus amino acid residues that differ from human G6Pase-α at more than 36 positions (Cao et al. 2021). Screening the top ten consensus residues-targeted hG6PC mRNA variants, each of which encodes a protein mutant with a single amino acid substitution, yielded three hG6Pase-α substitutions with improved protein expression and activity: a glutamine (Q) to arginine (R) substitution at positions 139 (Q139R) or 247 (Q247R), or a serine (S) to cysteine (C) substitution at position 298 (S298C) (Cao et al. 2021). Notably, the protein variant S298C was found to have > twofold increase in protein expression levels compared to wild-type human G6Pase-α (hG6Pase-α) (Cao et al. 2021), consistent with other reports (Zhang et al. 2019, 2020). In the present study, we sought to investigate the cellular and molecular mechanism(s) contributing to such an improvement. Our findings uncover a critical role that a single amino acid residue can play in determining the expression and stability of hG6Pase-α. Additionally, we have also studied the effect of the N-linked glycosylation on the expression and activity of hG6Pase-α in cell-based models.

## Materials and methods

### mRNA production

Complete N1-methylpseudouridine substituted mRNA was synthesized in vitro from a linearized DNA template containing the 5′ and 3′ untranslated regions (UTRs) and a poly-A tail, as previously described (Richner et al. 2017). After purification, the mRNA was diluted in citrate buffer to the desired concentration and frozen (An et al. 2018).

### Mammalian cell culture and transfection

HeLa cells were obtained from ATCC and maintained in DMEM media (10% FBS, v/v) at 37 °C supplied with 5% CO_2_. One day prior to transfection, 120,000 cells/well were seeded on 6-well plates, resulting in ∼50–70% confluency on the day of transfection. Cells were transfected with 0.5 µg of mRNA using Lipofectamine 2000 or Messenger MAX™ (Invitrogen) by following manufacturer’s protocol. At various time points (2–72 h) post-transfection, cells were harvested and used for protein expression or enzymatic activity measurements. Under certain circumstances, 6 h after transfection with indicated mRNA constructs, the cells were allowed to continue incubating for 16 h in the medium supplied with 0.1% (v/v) DMSO (vehicle) or lactacystin (EMD Millipore Corp. USA) at 10 µM before they were collected for protein expression analysis.

### Protein expression analysis

hG6Pase protein expression levels in cell lysates were measured by standard immune blotting procedure, using the LI-COR odyssey system. Total protein concentration of cell lysates were quantified by Pierce® BCA Protein Assay kit (Thermo Scientific). Samples were separated by 4–12% SDS-PAGE gel and transferred to nitrocellulose membranes by dry blotting system (iBlot2, Invitrogen). The target proteins, hG6Pase-α, ERP72, and β-actin were probed with primary antibodies anti-hG6Pase-α (1:500, HPA052324, Atlas Antibodies), anti-ERP72 (1:500, D70D12, Cell Signaling), and anti-β-actin (1:1000, 8H10D10, Cell Signaling), respectively, followed by incubation with (IR)-labeled goat anti-rabbit secondary antibody (1:5000, IRDye® 800CW, LI-COR). IR-intensity signals were detected and quantified by Odyssey CLx (LI-COR Biosciences).

### G6Pase activity assay

G6Pase enzymatic activity was measured by the release of inorganic phosphate from G6P using Taussky and Shorr’s method (Taussky and Shorr 1953). Briefly, in a round bottom 96-well plate, 40 µl of 200 mM G6P, 100–115 µl of 100 mM BIS–Tris buffer (pH 6.5) and 5–20 µl of transfected cell lysates were added and incubated at 37 °C for 30 min. Then, 40 µl of 20% (w/v) trichloroacetic acid (TCA) solution was added to each well and incubated at room temperature for at least 5 min to quench the reaction. Subsequently, the reaction mixture was centrifuged at 1,800 g for 20 min to sediment the precipitated protein and other debris. A portion of supernatant (25–50 µl) was transferred to a new transparent flat-bottom 96-well plate and mixed with 50–75 µl of distilled water and 100 µl of pre-made Taussky and Shorr’s color reagent containing 1% ammonium molybdate, 5% (w/v) Iron (II) sulfate, and 0.5 M sulfuric acid, followed by incubation at room temperature for 5 min. Color development in reactions was measured by absorbance at 660 nm and the released inorganic phosphate (Pi) was determined based on a series of Pi standards. Final G6Pase enzymatic activity was expressed as amount of Pi (nmol) released per mg of total protein per minute of reaction time (nmol/min/mg total protein). The total protein concentration in cell lysates was determined by the BCA assay as described above.

### Immunocytochemistry analysis

HeLa cells were plated in 96-well, glass bottom plates (655,892, Greiner Bio-One) using recommended culturing conditions, at a density of 15,000 cells per well. Cells were either kept non-transfected or transfected with the hG6PC mRNA or relevant variants (50 ng/well) using Lipofectamine 2000. At a predetermined time point (0–72 h) post-transfection, the cells were fixed in 4% (w/v) PFA, permeabilized in 0.5% (v/v) Triton X100, blocked in 1% (w/v) BSA and followed by immunofluorescent staining with anti-G6Pase rabbit Ab (1:50, HPA052324, Sigma) and anti-Calnexin mouse Ab (1:500, MA-15389, Thermo) to examine the ER localization. Secondary antibody incubation was used to amplify the signal (1:1000, goat anti-rabbit Alexa 488 and goat anti-mouse Alexa 647 respectively). The cells were counter stained with DAPI for nuclei visualization. For image acquisition and co-localization analysis, samples were imaged on the Opera Phenix spinning disk confocal microscope (Perkin Elmer), using a 63X water immersion objective (NA 1.15). 45 fields of view (~ 20 cells each) have been imaged for each sample. The hG6Pase was imaged with the 488-nm laser line, the ER marker Calnexin was imaged with the 647-nm laser line, and the nuclear stain was imaged with the 405-nm laser line. A z-stack of five optical sections spanning 2.5 µm were acquired for all three channels.

### Statistical analysis

All data are shown as means ± SEM. For statistical analysis, significance was determined using an unpaired *t*-test between different groups. A *p*-value of < 0.05 was statistically significant. *, **, ***, and **** were used to define significant levels at *p* < 0.05, *p* < 0.01, *p* < 0.001, and *p* < 0.0001, respectively.

## Results

### Characterization of the hG6Pase-α variants with enhanced cellular expression by site-specific alanine substitution and codon choice

**Fig. 1:**
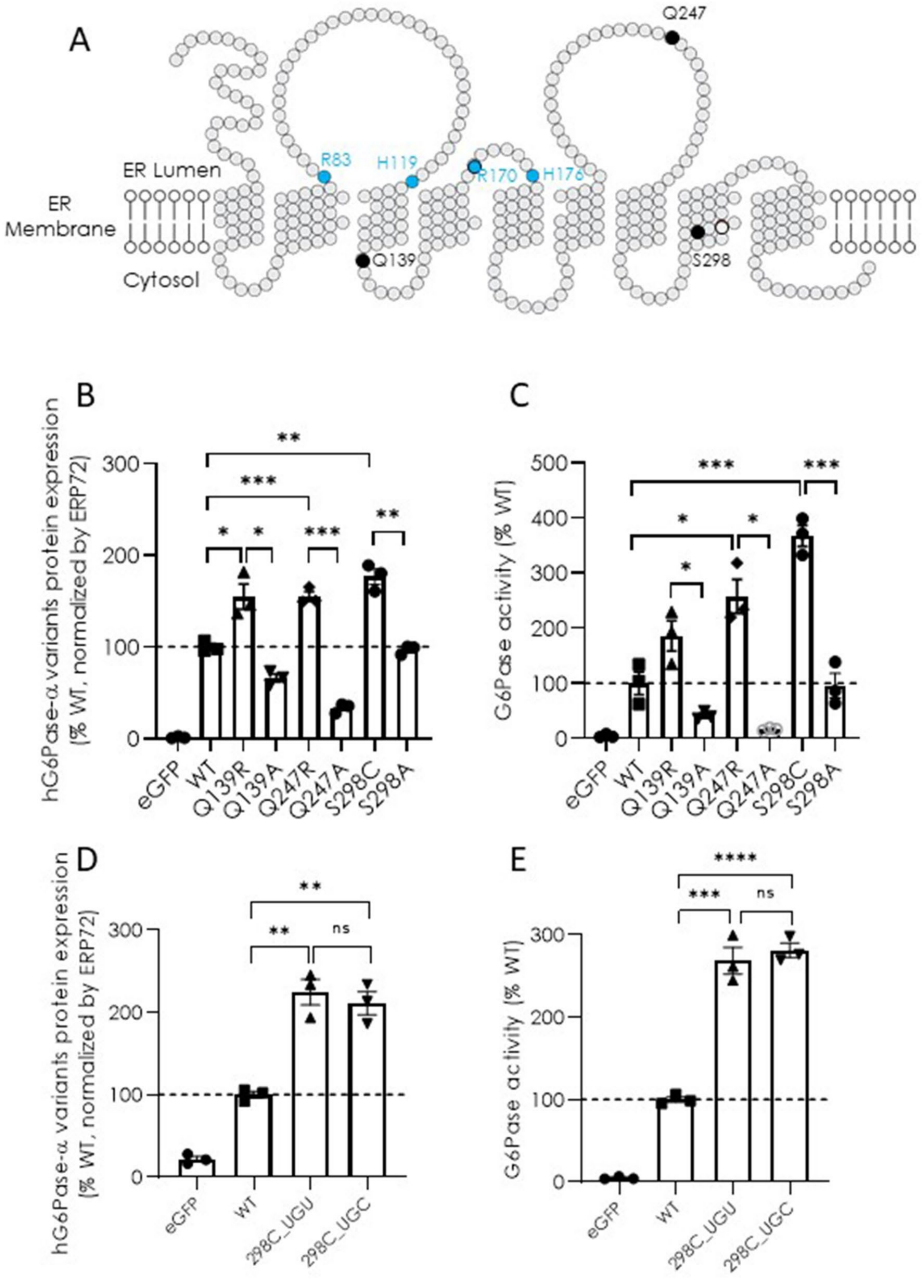
Protein expression and G6Pase enzymatic activity of hG6Pase-α variants directed by hG6PC mRNA constructs. **A** Topological analysis of hG6Pase-α shows a nine-transmembrane spanning ER enzyme. Black dots: locations of Q139, Q247, and S298 for targeted substitutions. Blue dots: residues directly involved in enzymatic activity. **B and C** protein expression (**B**) and G6Pase enzymatic activity (**C**) of hG6Pase-α variants encoded by hG6PC mRNA constructs encoding wild-type protein (WT) or protein variants containing a point mutation as indicated. **D and E** protein expression (**D**) and G6Pase enzymatic activity (**E**) of WT and the S298C hG6Pase-α variants encoded by two different codons at the mutated site. The mRNA constructs were transfected into HeLa cells and examined for protein expression and G6Pase activity in cell lysates at 48-h post-transfection, as described under “Materials and methods”. For protein expression analysis, the quantified signals from the hG6Pase-α variants were also normalized by Erp72, a house-keeping ER marker protein. Data were shown as percentage of wild-type (WT) group and presented as mean ± SEM of *n* = 3 samples from independent transfections. *p < 0.05; **p < 0.01; ***p < 0.00, ****p < 0.0001. *ns* non-significant

Using a bioinformatics-aided approach, we have previously identified three hG6Pase-α variants with improved protein expression, each of which carries a single amino acid substitution: Q139R, Q247R, and S298C (Cao et al 2021). A closer look at the sequence alignment of human G6Pase-α and nineteen other mammalian homologues reveals that all mammalian G6Pases except for those of human and a limited number of non-human primates encode the three consensus amino acids that confer improved protein expression (Supplementary Fig. 1, boxed). Predicted topological analysis of hG6Pase-α (Fig. 1a) indicate that all three positions (marked as blacked dots) are located away from the known catalytic center of hG6Pase-α (R83, H119, R170, and H176, marked as blue dots). To further examine the specificity of the benefit of these substitutions, we generated mRNA mutants in which an alanine was substituted at each of the corresponding positions (i.e., Q139A, Q247A, or S298A), and tested their expression and activity in transfected cells versus wild-type (WT) and the original consensus mutants. Our results show that the alanine substitutions completely abolished any gains in protein expression and activity conferred by the consensus substitutions (Fig. 1b and 1c), confirming the specificity of benefit of these consensus substitutions. Based on the levels of improvement, the increase in activity conferred by Q139R, Q247R, or S298C is presumably due to an elevated level of protein expression. Notably, since S298C showed the greatest increase in both protein expression and activity (Fig. 1b and 1c) we have focused on this variant in the remainder of this report. Since cysteine is encoded by the UGU and UGC codons, and different codon usage may lead to a change in protein expression (Kudla et al. 2009), we next tested whether a synonymous codon substitution for cysteine at position 298 (298C) has any impact. Our results show that the UGU- or UGC-encoded 298C resulted in similar improvements in protein expression and activity compared to the hG6PC mRNA encoding wild-type amino acid sequence when transfected in HeLa cells (Fig. 1d and 1e), suggesting that the choice of cysteine-encoding codon at this position does not affect protein expression.

### Characterization of expression of hG6Pase-α variants at S298 in a cell-free translating system

We next measured protein expression of hG6PC mRNA variants in an in vitro cell-free translating (CFT) system to test whether the 298C substitution alters protein translation in a cell-free environment. Our data clearly show that WT and mutant constructs with either enhanced (S298C) or unchanged (S298A) capability in protein expression in cultured cells yielded nearly identical levels of protein expression in the CFT system (Supplementary Fig. 2), suggesting that the impact of amino acid variations at the position 298 on expression are dependent on the integrity of the cells or environment inside the living cells.

### Identification and characterization of hG6Pase-α variants at S298 with marked reduction in cellular expression

**Fig. 2:**
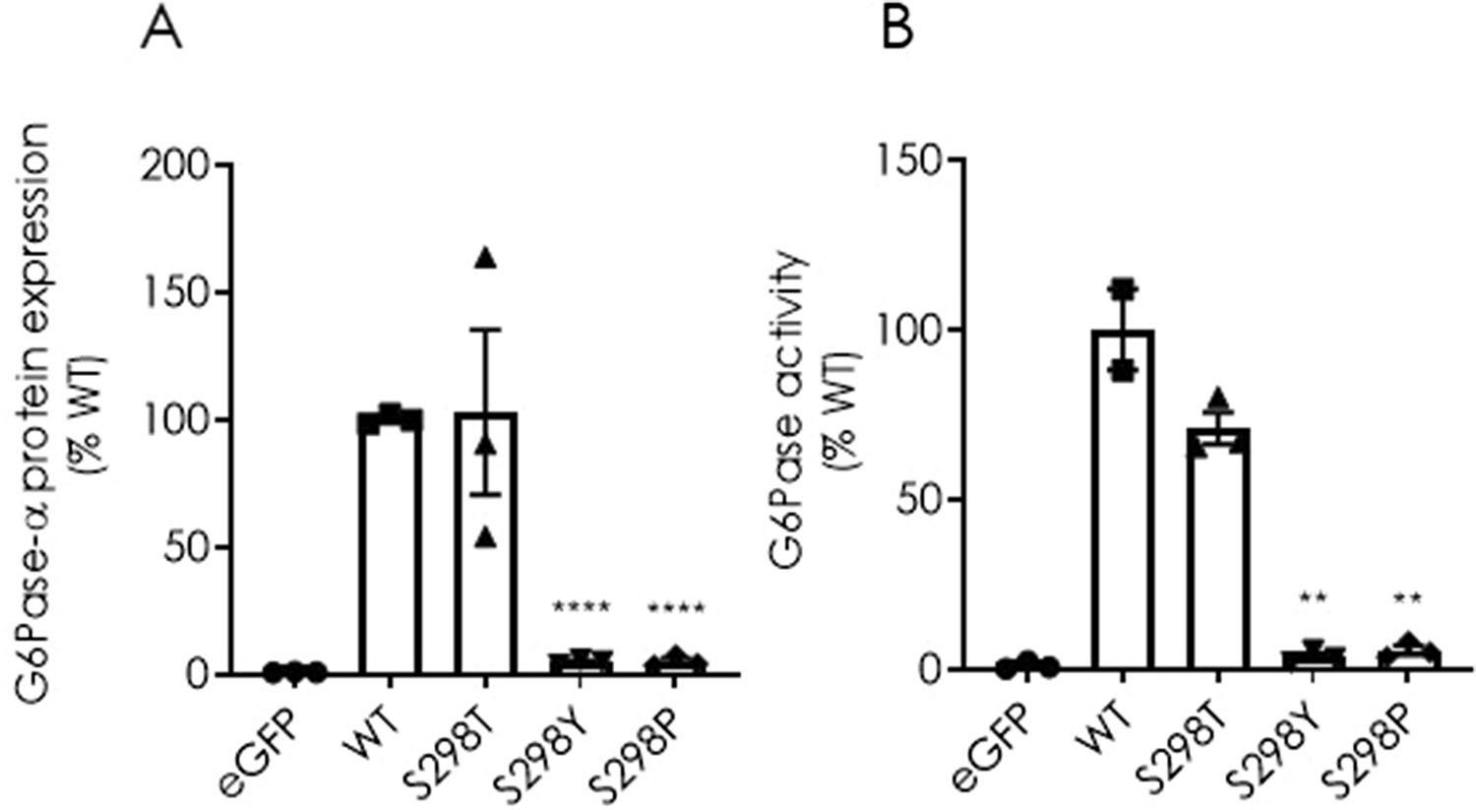
Protein expression (**A**) and G6Pase enzymatic activity (**B**) of hG6Pase-α variants directed by wild-type (WT) or hG6PC mRNA variants with additional targeted mutations at Ser-298. The experiments were performed as described under “Materials and methods” and legend for Fig. 1. Data were shown as percentage of wild-type (WT) group and presented as mean ± SEM of n = 3 samples from independent transfections. **p < 0.01; ****p < 0.0001 vs. WT

While the S298C substitutions yielded a significant increase in protein expression and activity, a proline substitution mutant at the same position (S298P) has been previously identified as a loss-of-function mutation that causes GSD1a disease (Shieh et al. 2002). This is very intriguing since the S298 resides within the eighth transmembrane domain of hG6Pase-α that is not directly associated with its catalytic activity (Fig. 1a). To gain further insights on the impact of different amino acid residues at position 298 on hG6Pase-α protein expression and enzymatic activity, we next tested threonine (T), tyrosine (Y), or proline (P) substitution variants in transfected HeLa cells. The T and Y substitutions were tested mainly because they share physicochemical properties with S. While the S298T substitution had no impact on protein expression and enzymatic activity compared to that of WT, a dramatic decrease in both measurements was observed with the S298Y or S298P substitutions (Fig. 2a and 2b). Again, the reduction in activity is presumably due to a reduced level of protein production for Y or P substitutions.

### Kinetic analysis of cellular expression and localization of hG6Pase-α variants

**Fig. 3:**
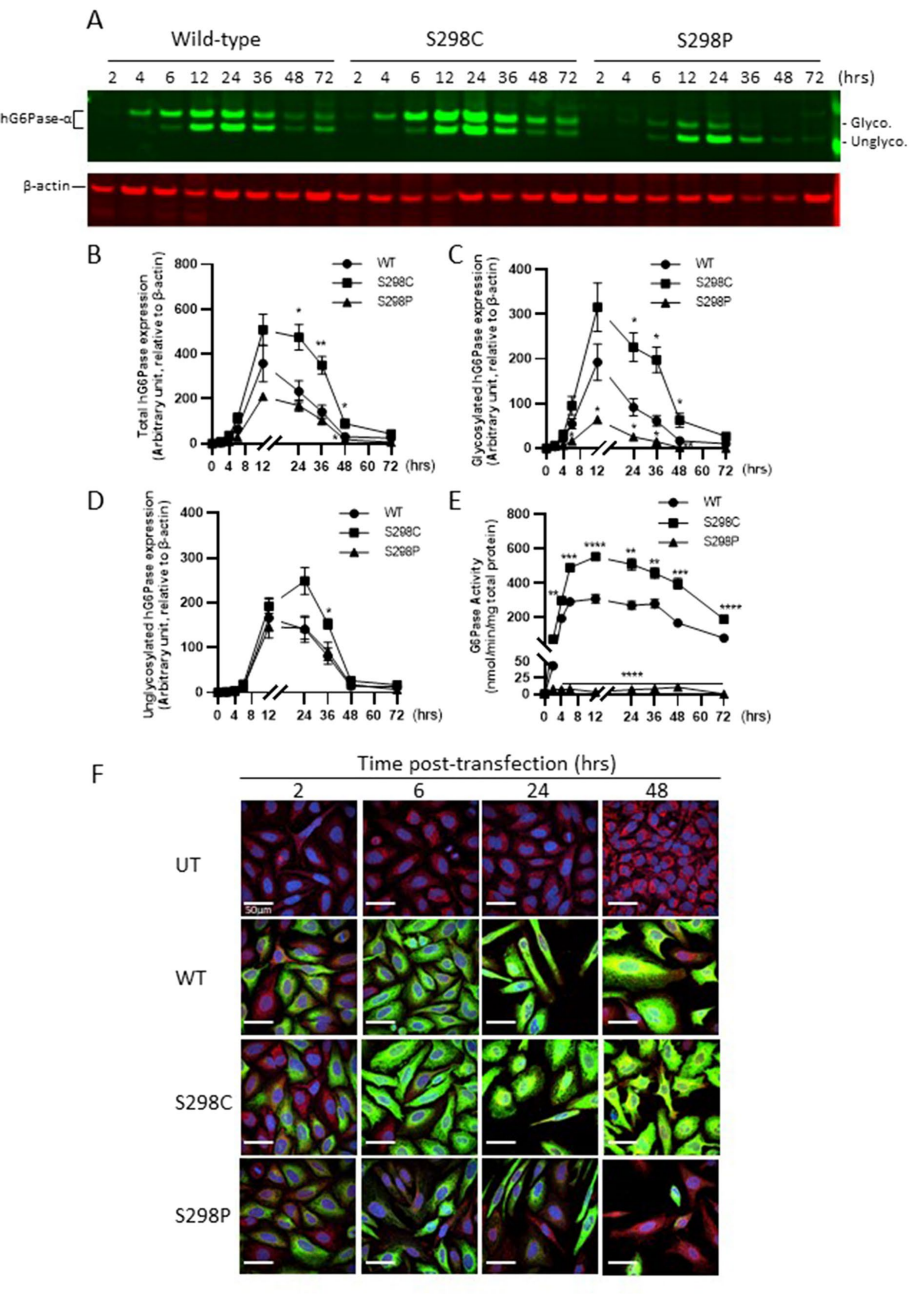
Kinetic analysis of protein expression (**A-D, F**) and G6Pase enzymatic activity (**E**) of wild-type (WT) hG6Pase-α and its variants harboring S298C or S298P mutation in mammalian cells transfected with hG6PC mRNA constructs. The mRNA constructs were transfected into HeLa cells and examined for protein expression and G6Pase activity in cell lysates at different time (2 to 72 h) post-transfection. Panel **A** represents a western blotting analysis of wild-type hG6Pase-α and variants harboring the S298C or S298P mutations, which migrated as two polypeptides (non-glycosylated or glycosylated), especially at the time points between 6 and 48 h, post-transfection. For protein expression analysis in panels **B** to **D**, the quantified signals from the hG6Pase-α variants were normalized by beta-actin. Panel **E** shows the G6Pase enzymatic activity in cell lysates prepared at different time points post-transfection. **F** kinetic analysis of subcellular expression and localization of wild-type (WT) hG6Pase-α and its variants harboring S298C or S298P. HeLa cells were transfected with mRNA constructs and analyzed for subcellular expression of hG6Pase-α and variants with immune-cytofluoresence-based confocal microscopy. Green: hG6Pase-α and its variants, Red: Calnexin, an ER marker. The cells were also counterstained with DAPI (blue) for nucleus visualization. All samples were stained for hG6Pase-α, Calnexin, and DAPI. Scale bars are 50 µm. All quantitative data are presented as mean ± SEM of *n* = 3 samples from independent transfections. *p < 0.05; **p < 0.01; ***p < 0.001; ****p < 0.0001 vs. WT at the same time point. Data shown on panel **F** are representative of at least two independent experiments. *Hrs* hours, *UT* untransfected

Our results demonstrate that a single amino acid substitution at position 298 of hG6Pase-α could have a substantial impact on its expression in mammalian cells. To further examine the effect of these variants, we next performed a time-course study in which protein expression and enzyme activity in transfected cells were monitored for a wide range of post-transfection time points (i.e., 2, 4, 6, 12, 24, 36, 48, and 72-h) since our previous studies were mainly focused on 24–48 h. Consistent with previous reports (Pan et al. 1998b; Shieh et al. 2002), immunoblot analysis showed that the newly synthesized hG6Pase-α migrated on the SDS-PAGE gel as two bands with molecular masses of ~ 36- and ~ 32-kDa, representing glycosylated and non-glycosylated forms, respectively (Fig. 3a). hG6Pase-α protein expression and activity could be detected as early as 2-h post-transfection, reaching a peak at 12–24 h, and dropping to a minimal level at 72 h (Fig. 3a–e). Quantitative analysis of each of the two forms in this kinetic study also shows that the enhancement or reduction of protein production by S298C or S298P was observed at all time points; however, the divergence appears more prominent between 12 to 36 h post-transfection. More importantly, our data strongly suggest that the amino acid substitutions at position 298 had a more profound effect on the glycosylated versus non-glycosylated form (Fig. 3a, 3c and 3d). Our results also show that G6Pase activity in cells transfected with the S298P variant was minimal or undetectable despite a significant level of newly synthesized protein mainly in its non-glycosylated form (Fig. 3a, 3d and 3e).

Since hG6Pase-α is an ER-anchored membrane protein with 9 transmembrane alpha helices (Pan et al. 1998a) (Fig. 1a), we next investigated the subcellular localization of hG6Pase-α protein variants to learn whether the S298C and S298P mutations cause any alteration in ER localization. Confocal microscopy analysis shows that the newly synthesized hG6Pase-α WT and the two variant proteins encoded by modified mRNAs were localized to the ER since their immunostaining signals (in green) overlaid well with Calnexin, an ER marker (in red) (Fig. 3f). In contrast to the high levels of protein expression and ER localization of WT and S298C variants throughout the course of study (Fig. 3f, middle panels), the S298P protein variant shows a substantial loss of ER protein expression especially at late time points (i.e., 24 or 48 h) despite a comparable level of protein expression at early time points (i.e., 2 or 6 h) (Fig. 3f, bottom panel).

### Analysis of expression and activity of additional hG6Pase-α mutants targeting S298

To further evaluate the specific contribution of cysteine at position 298 on enhanced protein expression, this residue was further modified to amino acids with diverse physicochemical properties, including hydrophobic amino acids such as isoleucine (I), methionine (M), tryptophan (W), and phenylalanine (F), histidine (H), and hydrophilic amino acids such as aspartic acid (D), asparagine (N), and arginine (R), and assessed for their impact on protein expression and activity in cells transfected with the corresponding mRNA constructs. We have also tested cysteine substitutions at other positions including neighboring S297 and I299, and other native serine (positions 15, 85, 278, and 356). The newly synthesized hG6Pase-α variants appeared as both glycosylated and non-glycosylated forms in transfected HeLa cells at 24-h post-transfection (we chose this time point based on a high level of protein expression as shown in Fig. 3), so we analyzed protein expression for each form individually as well as in total and a ratio of the two forms (Table 1). The results clearly showed that the cysteine substitution at position 298 is unique in conferring enhanced protein expression (and activity) since: (1) none of the other mutations at the position 298 resulted in enhanced protein expression and activity; to the contrary, similar to S298P and S298Y as shown in Fig. 2, the majority of substitutions led to substantial reduction or loss of protein expression (especially in the glycosylated form) and G6Pase activity; (2) cysteine substitution at neighboring positions 297 or 299 did not confer increased protein expression and activity; and (3) cysteine substitutions at selected native serine sites across the entire molecule (positions 15, 85, 278, and 356) also failed to increase protein expression. Interestingly, it appears that the mutations at position 298 have a more profound effect on the newly synthesized hG6Pase-α in glycosylated form versus the non-glycosylated form, and the changes in activity levels are closely correlated with glycosylated, but not non-glycosylated proteins (Table 1). Additionally, the mutation of the N-glycosylated site at Asn96 to alanine (N96A) completely abolished glycosylation and rendered the protein inactive despite a large amount of non-glycosylated protein being synthesized (Table 1).

**Table 1:** Analysis of protein expression and enzymatic activity of hG6Pase mutants encoded by mRNA constructs

hG6PC mRNA_variants	Protein expression (% of WT)			Ratio (Glyco./Unglyco.)	G6Pase activity (% of WT)	Mutant amino acid classification
	Glyco	Nonglyco	Total			
WT	100.0 ± 6.0	100.0 ± 15.9	100.0 ± 8.2	1.9 ± 0.3	100 ± 21.6	–
S298C	172.4 ± 3.9	94.9 ± 11.8	145.3 ± 6.6	3.4 ± 0.3	194.7 ± 6.5	–
S298I	61.9 ± 8.8	82.9 ± 10.7	72.4 ± 9.3	0.7 ± 0.1	34.9 ± 5.3	Hydrophobic
S298M	29.8 ± 3.4	76.2 ± 5.8	53.1 ± 4.4	0.4 ± 0.0	6.5 ± 0.2	
S298W	28.0 ± 2.4	103.2 ± 8.7	65.7 ± 5.6	0.3 ± 0.0	0.80 ± 0.3	
S298F	10.0 ± 0.3	41.2 ± 8.7	17.3 ± 2.1	2.2 ± 0.5	8.3 ± 2.1	
S298H	16.9 ± 1.0	154.2 ± 1.1	64.8 ± 0.5	0.2 ± 0.0	3.6 ± 0.6	Neutral
S298D	19.5 ± 2.5	177.0 ± 20.6	74.4 ± 8.8	0.2 ± 0.0	1.2 ± 0.0	Hydrophilic
S298N	108.2 ± 4.9	156.9 ± 16.2	125.2 ± 7.3	1.3 ± 0.1	86.4 ± 10.0	
S298R	41.3 ± 2.0	101.7 ± 8.4	71.6 ± 3.5	0.4 ± 0.0	20.0 ± 2.5	
S297C	96.8 ± 10.4	61.8 ± 7.3	87.1 ± 8.1	3.3 ± 0.4	85.2 ± 11.9	Cysteine substitution for neighbor residues
I299C	48.5 ± 5.7	138.8 ± 10.5	80.0 ± 6.4	0.6 ± 0.1	34.9 ± 4.9	
S15C	57.1 ± 5.5	33.0 ± 0.2	50.0 ± 3.8	4.1 ± 0.8	175.2 ± 11.6	Cysteine substitution for other Serine residues
S85C	48.7 ± 4.8	66.4 ± 18.3	53.9 ± 8.0	1.8 ± 0.4	47.9 ± 8.5	
S278C	39.2 ± 3.1	83.0 ± 8.4	59.8 ± 5.0	0.5 ± 0.0	46.1 ± 5.1	
S356C	64.4 ± 5.4	67.8 ± 14.2	65.4 ± 7.5	2.3 ± 0.4	63.6 ± 2.8	
N96A	–	217.3 ± 33.6	109.0 ± 16.9	0.0 ± 0.0	0.9 ± 0.8	Disruption of glycosylated N96

hG6PC mRNA constructs encoding wild-type (WT) or mutant hG6Pase-α were transfected into HeLa cells. Twenty-four hours after transfection, protein expression and G6Pase activity in cell lysates were analyzed by Western blotting and release of inorganic phosphate from glucose-6 phosphate as described under “Materials and methods”. The ratio of glycosylated (Glyco.) versus unglycosylated (Unglyco.) hG6Pase-α variants was determined by the band intensity of each protein form from the same lane on the western blotting membrane. Protein expression and G6Pase activity data were shown as percentage of WT and presented as mean ± SEM of *n* = 3 samples from independent transfections

### Effect of cysteine-targeted mutations on the expression and activity of hG6Pase-α S298C variant

**Fig. 4:**
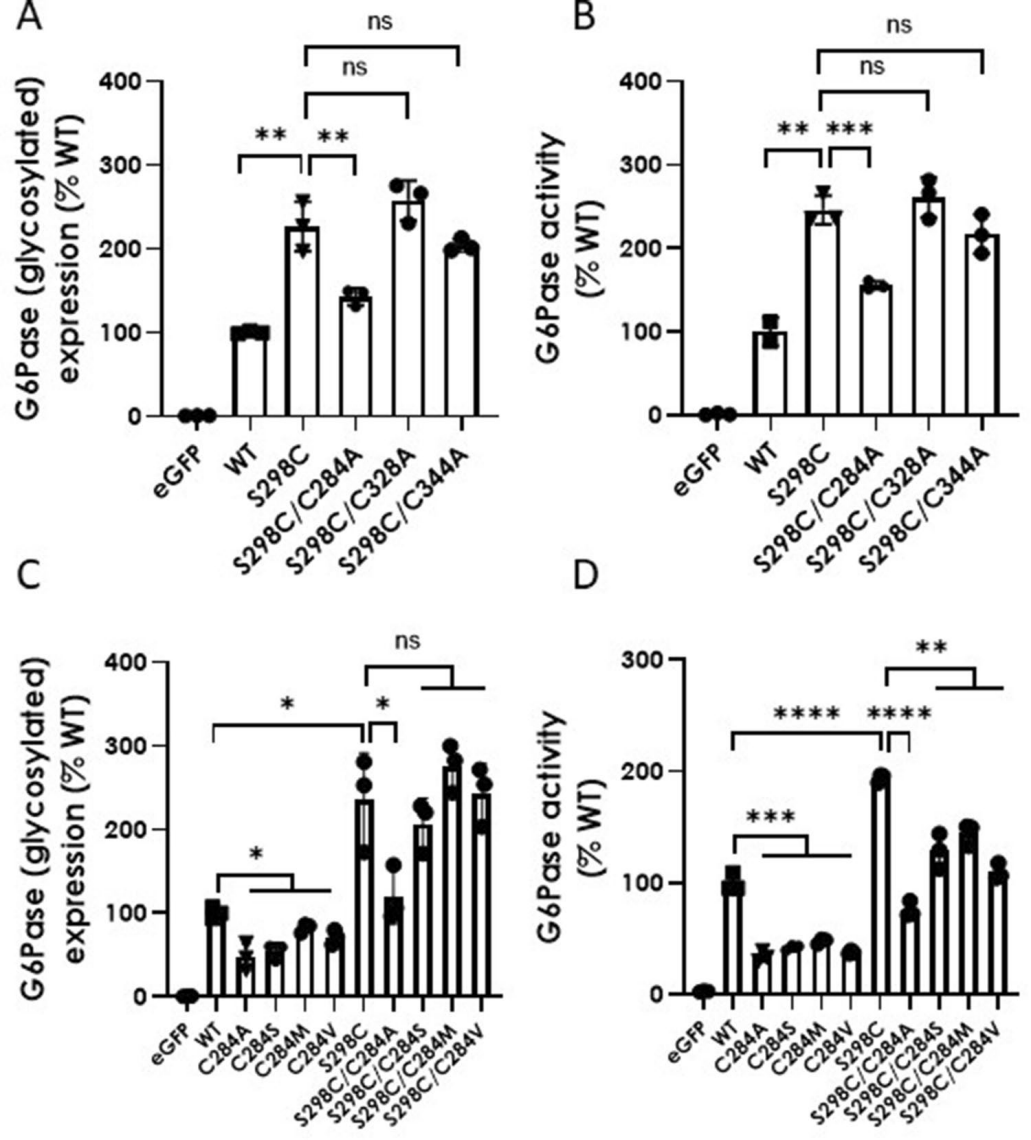
Protein expression (**A** and **C**) and G6Pase enzymatic activity (**B** and **D**) of wild-type (WT) hG6Pase-α and its variants targeted for selective substitution of cysteine. The mRNA constructs were transfected into HeLa cells and examined for protein expression of glycosylated hG6Pase-α and its variants and G6Pase activity in cell lysates at 24-h post-transfection, as described under “Materials and methods”. For protein expression analysis, the quantified signals from the glycosylated hG6Pase-α or its variants were also normalized by Erp72. Data were shown as percentage of WT group and presented as mean ± SEM of *n* = 3 samples from independent transfections. *p < 0.05; **p < 0.01; ***p < 0.001; ****p < 0.0001. *ns* non-significant

The introduction of an additional cysteine in the hG6Pase-α S298C variant raised the possibility that it may partner with another cysteine within the enzyme and form a new disulfide bond, which might facilitate the folding process of the newly synthesized protein and make it more stable. To explore this possibility, we produced hG6PC mRNA constructs carrying dual or paired mutations in which another cysteine of interest was mutated to alanine or another amino acid without a free sulfhydryl (SH) group on the S298C backbone and compared their expression and activity with the hG6Pase-α variant carrying the single S298C mutation. To this end, we have focused on three cysteine sites that are possibly in proximity to 298C based on topology analysis (Supplementary Fig. 3a) and structural prediction (Supplementary Fig. 3b) (https://alphafold.ebi.ac.uk/entry/P35575): C284, C328, and C344. We have also focused our protein expression analysis on the glycosylated hG6Pase-α form since this is the predominant form and contributes the majority (if not all) of the G6Pase activity introduced into the transfected HeLa cells via WT or the S298C mutant (Fig. 3a–e and Table 1). As shown in Fig. 4a and 4b, in comparison with the S298C single mutant, the mRNA construct carrying the S298C/C284A pair mutations was identified as the only candidate resulting in a significant reduction in both protein expression and G6Pase activity. However, the C284A mutation alone also led to decreased protein expression and G6Pase activity when compared with the WT construct (Fig. 4c and 4d), obscuring the potential impact of a loss of disulfide bonding between 298 and 284C. Therefore, we have generated additional mutants focused on C284 with both WT and S298C backgrounds and examined their protein expression and activity (Fig. 4c and 4d). The pair mutations (S298C/C284S, S298C/C284M, and S298C/C284V) did not result in significantly reduced protein expression in comparison to the S298C mutation alone (Fig. 4c and 4d). The observed decrease in activity in pair mutations versus S298C mutation alone is expected to be attributed to a reduction in intrinsic activity of these C284 mutants, which also caused a reduction in activity levels when compared with WT control (Fig. 4c and 4d). Collectively, the data do not lend sufficient biochemical evidence supporting the role of a potential disulfide bond between 298C and proximate cysteine at sites 284, 324, or 344.

### Effect of lactacystin, a proteasome inhibitor, on the expression of hG6Pase-α protein

**Fig. 5:**
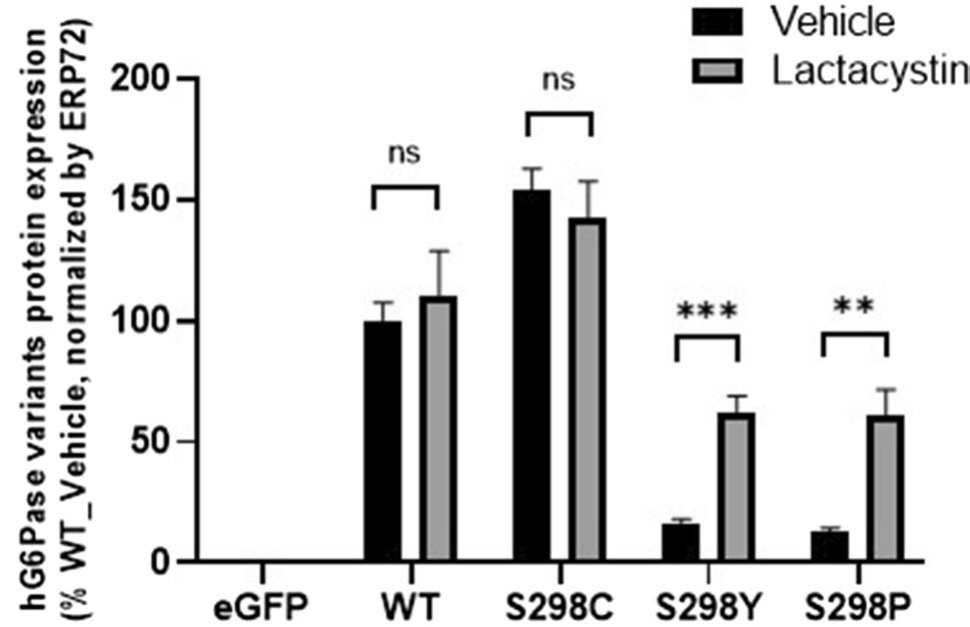
Effect of proteasome inhibitor, lactacystin, on protein expression of hG6Pase-α and its variants. HeLa cells were first transfected with mRNA constructs. At 6 h post-transfection, medium was replaced with that containing vehicle (0.1%, v/v, DMSO) or lactacystin at 10 µM and the cells were incubated for additional 16 h before they were collected for protein expression analysis. The graph shows the analysis of glycosylated hG6Pase-α and its variants. Data were presented as mean ± SEM of n = 3 samples from independent transfections. **p < 0.01; ***p < 0.001 vs. vehicle. *ns* non-significant

As an ER-localized integral membrane protein with multiple transmembrane domains, the newly synthesized mammalian G6Pase is expected to follow the co-translational translocation pathway to insert into the ER membrane (Shao et al. 2017), and to undergo a quality control process to ensure that only properly folded protein resides in the ER. The incorrectly folded protein is removed by an ER-associated protein degradation (ERAD) pathway, in which the protein will be pulled into the cytosol, poly-ubiquitinated, and degraded by the proteasome (Wu and Rapoport 2018). We hypothesized that the amino acid substitutions, especially those in the transmembrane domains including the position 298, might affect protein folding. For instance, the S298P and S298Y variants might not reach a correctly folded state resulting in rapid degradation. To test this hypothesis, we used a proteasome inhibitor lactacystin (Fenteany et al. 1995). Treatment of transfected cells with lactacystin significantly increased the levels of glycosylated S298P and S298Y hG6Pase variants (Fig. 5), suggesting an active proteasome-mediated degradation process for these variants, which causes a dramatic reduction in protein expression in comparison with the WT and S298C variant.

## Discussion

One advantage of mRNA-based therapies for treating or preventing human diseases is that mRNA molecules may be modified chemically or at the sequence levels to improve protein translation, extend half-life and stability, and enhance the intrinsic activity of the targeted protein. To this end, we have employed bioinformatics approaches coupled with cell-based and in vivo screening and identified a sequence-engineered hG6PC mRNA encoding the protein variant S298C that leads to enhanced protein expression and is more efficacious than the wild-type sequence in a GSD1a mouse model (Cao et al. 2021). In contrast, tyrosine (Y) or proline (P) substitutions at site 298 led to substantial losses of both protein expression and G6Pase activity. Therefore, incorporation of different amino acid residue at site 298 had a significant effect on hG6Pase-α expression, ranging from a total loss of protein to more than a twofold increase in comparison with the native serine. Similarly, our study shows that amino acid substitutions in other transmembrane helices also caused substantial changes in protein expression. Furthermore, a time-course analysis on newly synthesized hG6Pase-α variants showed that the amino acid substitutions at site 298 have a more profound effect on the mature and fully active glycosylated form versus the nascent and less active non-glycosylated form. Confocal imaging analysis of newly synthesized hG6Pase protein in the ER revealed that the changes in protein expression of targeted variants was more pronounced at the late, but not the early, post-transfection time points. Collectively, these data strongly suggest that amino acid variations at site 298 may not affect protein translation per se; rather these substitutions may alter protein folding and subsequently affect stability in the ER once the protein is produced. A cysteine at this site may improve protein folding; while proline, tyrosine, and phenylalanine substitutions might affect the structural integrity of the newly synthesized protein, leading to misfolding and subsequent retro-translocation into the cytosol for degradation by ERAD (Wu and Rapoport 2018). Consistent with this notion, our data demonstrate that small molecule inhibition of proteasome by lactacystin improved expression of those unstable hG6Pase-α variants. Additionally, residue 298 is embedded inside a transmembrane helix domain and away from the catalytic center located inside the ER lumen, and thus was not expected—and does not appear—to affect the intrinsic activity of hG6Pase-α.

Notably, S298P is one of many naturally occurring mutations of hG6Pase that causes GSD1a in humans (Shieh et al. 2002; Chou et al. 2017). Our data presented in this study, together with previous reports (Shieh et al. 2002), suggest a novel molecular mechanism for genetic diseases like GSD1a. Instead of causing the total loss of enzymatic activity or a protein truncation, S298P, which is in a transmembrane helix, may result in a misfolded and unstable protein being rapidly degraded after synthesis. Many other disease-causing mutations in the transmembrane helices of hG6Pase lead to significant reduction in protein expression (Shieh et al. 2002). Conversely, S298C was identified as a substitution that significantly increases protein expression. Further mechanistic studies pertaining to the impact of the amino acid residues at this site and others on transmembrane helices of hG6Pase-α will not only help elucidate the molecular genetics of GSD1a (e.g., the genotype–phenotype relationships, and the heterogeneity of the clinical presentation of GSD1a), but also provide additional protein engineering options for developing candidate gene- and/or mRNA-based therapies for GSD1a.

Our alignment analysis of G6Pase-α homologues at position 298 (hG6Pase-α) revealed that the serine is a rare residue only found in human and in a limited number of non-human primates, while the cysteine is a consensus residue commonly found in most mammalian species including mouse, rat, canine, and many non-human primates (Cao et al. 2021). This suggests that the sequence divergence may have happened during a late phase of mammalian evolution. Though the introduction of the S298 substitution appears to be an unfavorable event for primates since it reduces protein stability/expression, this switch apparently was still sufficient in maintaining euglycemia and other essential biochemical and physiological functions for G6Pase-α in affected primates. In support of this notion, GSD1a is an autosomal recessive disorder and the heterozygous deletion or disruption of *G6pc* in mice does not cause any known symptoms of GSD1a (Lei et al. 1996), so that less than 50% of normal level of G6Pase activity is needed for maintaining a disease-free state in both human and mouse. Although it is still premature to firmly establish a minimal expression and/or activity threshold needed to rescue GSD1a, accumulated data from other groups and our own studies suggest that only 3–5% of normal hepatic G6Pase activity is needed to maintain glucose homeostasis and lower the risk of liver tumor development (Lee et al. 2015; Kim et al. 2017) (unpublished data).

Our mutagenesis studies at S298 and adjacent sites (i.e., S297 and I299) suggest that the introduction of cysteine at site 298 is unique in enhancing protein expression since 1) introduction of other physiochemically-diverse amino acids at site 298 did not boost protein expression and 2) substitution of cysteine at S297 or I299 did not boost protein expression. This raises a possibility that the introduced C298 may partner with another cysteine and form a new disulfide bond, which may subsequently stabilize the protein. As an integral membrane protein anchored in the ER by multiple transmembrane helices, hG6Pase-α is likely synthesized by the classical co-translational translocation pathway, in which protein synthesis and localization into ER happens simultaneously (Rapoport 1990; Shao and Hegde 2011). This co-translational pathway is defined by recognition of the first hydrophobic segment of the newly formed polypeptide by the signal recognition particle (SRP), followed by insertion into or translocation across the membrane by the Sec61 translocon channel (Voorhees and Hegde 2016). Based on this model, we hypothesized that C298, located in the eighth transmembrane helix, might interact with C284, C328, or C344 during protein synthesis or folding process and form a disulfide bond. To test this hypothesis, we first generated hG6Pase-α mutants harboring both S298C and a cysteine to alanine substitution at site 284, 328, or 344 and compared their protein expression levels with the S298C single mutant. Although our initial experiments suggested C284 as a potential disulfide partner, additional mutagenesis studies (C284S, C284M, or C284V) at this site were not consistent with disulfide bond formation between C298 and C284. Collectively our data suggest that intramolecular disulfide bond formation is less likely. However, it has been reported that a disulfide bond between the synthesized polypeptide and the translocon itself can stabilize the protein (Cannon et al. 2005). It remains unclear whether the introduced C298 forms a disulfide bond with a cysteine within translocon Sec61 or another unknown protein. In addition, the cysteine residue is widely used for protein S-palmitoylation, a covalent and reversible attachment of a palmitic acid group via a thioester linkage that plays important roles in regulating protein trafficking, localization, stability, and interactions with other effectors (Aicart-Ramos et al. 2011; Main and Fuller 2021). The protein S-palmitoylation is catalyzed by a group of enzymes called palmitoyl acyltransferases (PATs), many of which are also located in ER and/or Glogi (Zhang et al. 2021). Future work should focus on evaluating whether the C298 of mammalian G6Pase-α is palmitoylated, which may, at least in part, contribute to the observed improvements in protein folding and stability.

In summary, we have investigated the cellular and molecular mechanism(s) contributing to protein expression and activity of hG6Pase-α variants, especially for those substitutions at site of 298. Our findings uncover a critical role that a single amino acid residue plays in controlling the cellular level of newly synthesized hG6Pase-α. Our results also shed new insight into the molecular genetics of GSD1a, as well as demonstrate the power of protein engineering by harnessing these learnings to enhance and optimize candidate mRNA-based therapies to treat GSD1a or other devastating inherited metabolic disorders with limited treatment or management options.

## Supplementary Information

Below is the link to the electronic supplementary material.

Supplementary file1 (PDF 934 KB)

## Data Availability

The data that support the findings of this study are available from the corresponding authors upon reasonable request.

## Abbreviations

AHA: Azidohomoalanine
CFT: Cell-free translating
ER: Endoplasmic reticulum
ERAD: ER-associated protein degradation
ERT: Enzyme replacement therapy
G6P: Glucose-6 phosphate
G6Pase-α: Glucose-6-phosphatase-α
GSD1a: Glycogen storage disease 1a
HCAs/HCCs: Hepatocellular adenomas and carcinomas
PATs: Palmitoyl acyltransferases
Pi: Phosphate
SH: Sulfhydryl
S298C: Serine (S) to cysteine (C) substitution at the amino acid site 298
WT: Wild type
UTRs: Untranslated regions
