# Supplementary material for: Amnio acid substitution at position 298 of human glucose-6 phosphatase-α significantly impacts its stability in mammalian cells
Source: Amino Acids. 2023 Mar 21;55(5):695–708. doi: 10.1007/s00726-023-03263-8 (PMC10247848; doi:10.1007/s00726-023-03263-8)
Supplement: Supplementary file 1 — Supplementary file1 (PDF 934 KB) [file 726_2023_3263_MOESM1_ESM.pdf]

SUPPLEMENTARY MATERIALS

Title: Amnio acid substitution at position 298 of human glucose-6 phosphatase-α significantly impacts its stability in mammalian cells

Cao et al.

Cell-free in vitro translating assay

In vitro translation of G6Pase mRNA was accomplished using input concentrations of 0, 5, 10, 15, 20 and 25 ng/µL to a Wheat Germ Extract Kit (L4380, Promega) modified with L-Azidohomoalanine (C10102, Invitrogen), these concentrations are within the linear range for translation of G6Pase mRNA in this system. This was followed by a click reaction with a fluorescently labeled cyclooctyne (929-50000, LiCor), resuspended to 0.25 mM in DMSO. L-Azidohomoalanine was resuspended to 100 mM in nuclease free water. Kit components were combined into a cocktail at stock concentrations in the following volumes: wheat germ extract 43 µL, potassium acetate 7 µL, amino acid mixture (-) methionine 8 µL, azido homoalanine 4 µL, nuclease free water 18 µL. Reactions were initiated in a microplate by mixing in 5 µL of mRNA to 20 µL of cell-free cocktail with a pipette. The plate was then sealed, and reactions were allowed to proceed at room temperature for 2 hours in the dark. After 2 hours, 1 µL of fluorescent cyclooctyne was added, the plate was resealed, vortexed, spun down and allowed to proceed for another hour. Protein products were separated on a 4-12% bis-tris gel and visualized on the LI-COR odyssey system. Target proteins were quantified relative to a standard band present in lysate reactions with no added mRNA.

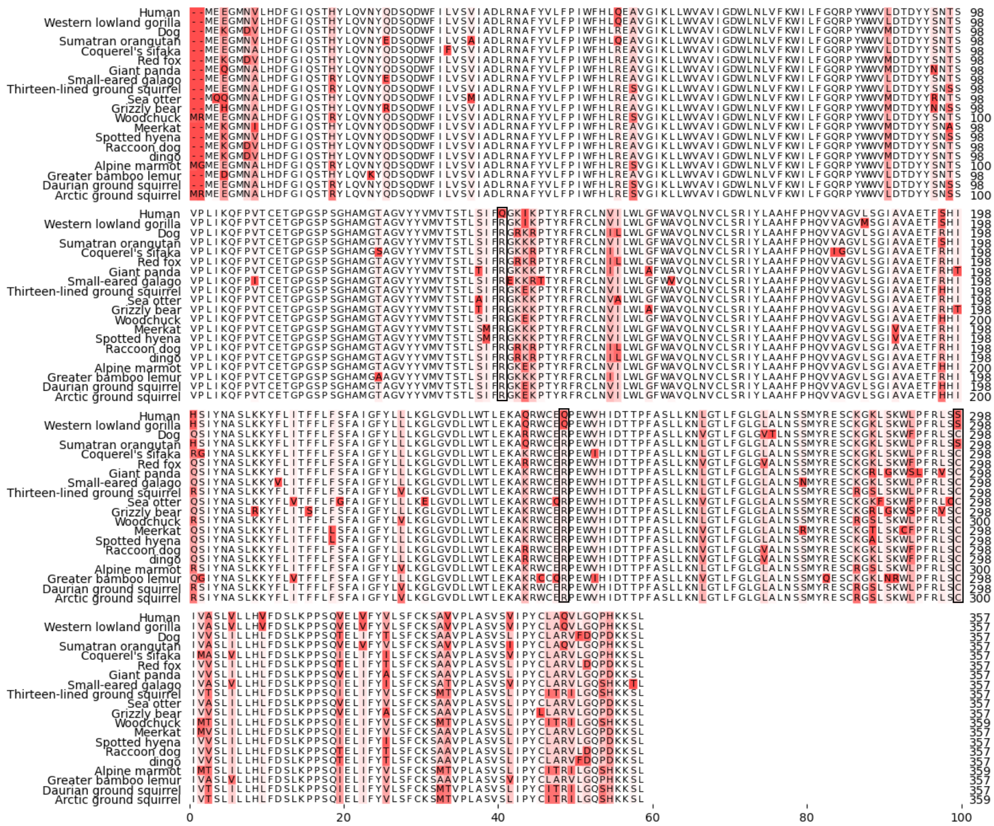

Supplementary Figure 1. Alignment of the amino acid sequences of some mammalian G6Pase-α. Note that Glutamine (Q) at positions of 139 or 247 and Serine (S) at position 298 (boxed) is only found in primates, while Arginine (R) and Cysteine (C) are consensus residues found in all other mammalian species as shown here. Residues at non-conserved positions are highlighted in color. The uniprot entry names for mammalian G6Pase-α sequences used for alignment are listed below in supplementary table 1 below.

Supplementary table 1. The uniprot entry names for the mammalian G6Pase-α sequences used for alignment analysis in supplementary Fig. 1

Uniprot Entry Name	Organism
G6PC1_HUMAN	Homo sapiens (Human)
G3RX42_GORGO	Gorilla gorilla gorilla (Western lowland gorilla)
G6PC1_CANLF	Canis lupus familiaris (Dog) (Canis familiaris)
A0A2J8REQ8_PONAB	Pongo abelii (Sumatran orangutan) (Pongo pygmaeus abelii)
A0A2K6GRZ5_PROCO	Propithecus coquereli (Coquerel's sifaka) (Propithecus verreauxi coquereli)
A0A3Q7RKW3_VULVU	Vulpes vulpes (Red fox)
D2I4E4_AILME	Ailuropoda melanoleuca (Giant panda)
H0X0J7_OTOGA	Otolemur garnettii (Small-eared galago) (Garnett's greater bushbaby)
I3MW65_ICTTR	Ictidomys tridecemlineatus (Thirteen-lined ground squirrel) (Spermophilus tridecemlineatus)
A0A2Y9K1A4_ENHLU	Enhydra lutris kenyoni (Sea otter)
A0A3Q7WTZ1_URSAR	Ursus arctos horribilis (Grizzly bear)
A0A5E4B9R5_MARMO	Marmota monax (Woodchuck)
A0A673V4H0_SURSU	Suricata suricatta (Meerkat)
A0A6G1AE98_CROCR	Crocuta crocuta (Spotted hyena)
A0A811XWX0_NYCPR	Nyctereutes procyonoides (Raccoon dog) (Canis procyonoides)
A0A8C0R019_CANLU	Canis lupus dingo (dingo)
A0A8C5YUE9_MARMA	Marmota marmota marmota (Alpine marmot)
A0A8C8YTA4_PROSS	Prolemur simus (Greater bamboo lemur) (Hapalemur simus)
A0A8C9PSF2_SPEDA	Spermophilus dauricus (Daurian ground squirrel)
A0A8D2HFR0_UROPR	Urocitellus parryii (Arctic ground squirrel) (Spermophilus parryii)

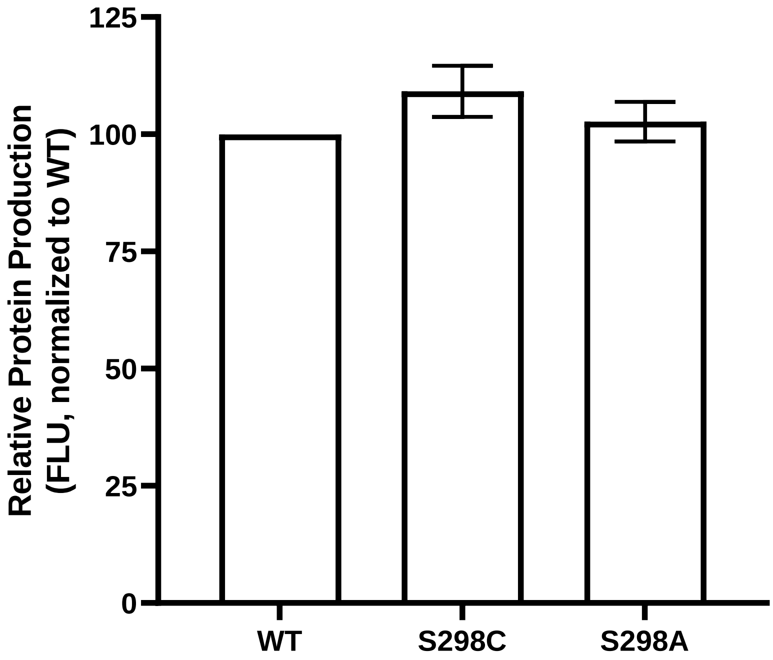

Supplementary Figure 2. Protein expression of hG6Pase-α and its variants analyzed by the cell-free *in vitro* translational assay. The assay was performed in an *in vitro* cell-free translation system containing wheat germ cell lysates, hG6PC mRNA variants, mixture of amino acids needed for protein translation with azidohomoalanine (AHA) as a surrogate for methionine. The newly synthesized proteins were separated by SDS-PAGE, visualized, and quantified as described in methods section. Each variant was run in triplicate and normalized to WT and averaged. Data were shown as percentage of Plotted average values and standard deviation were then calculated from the five normalized averaged levels.

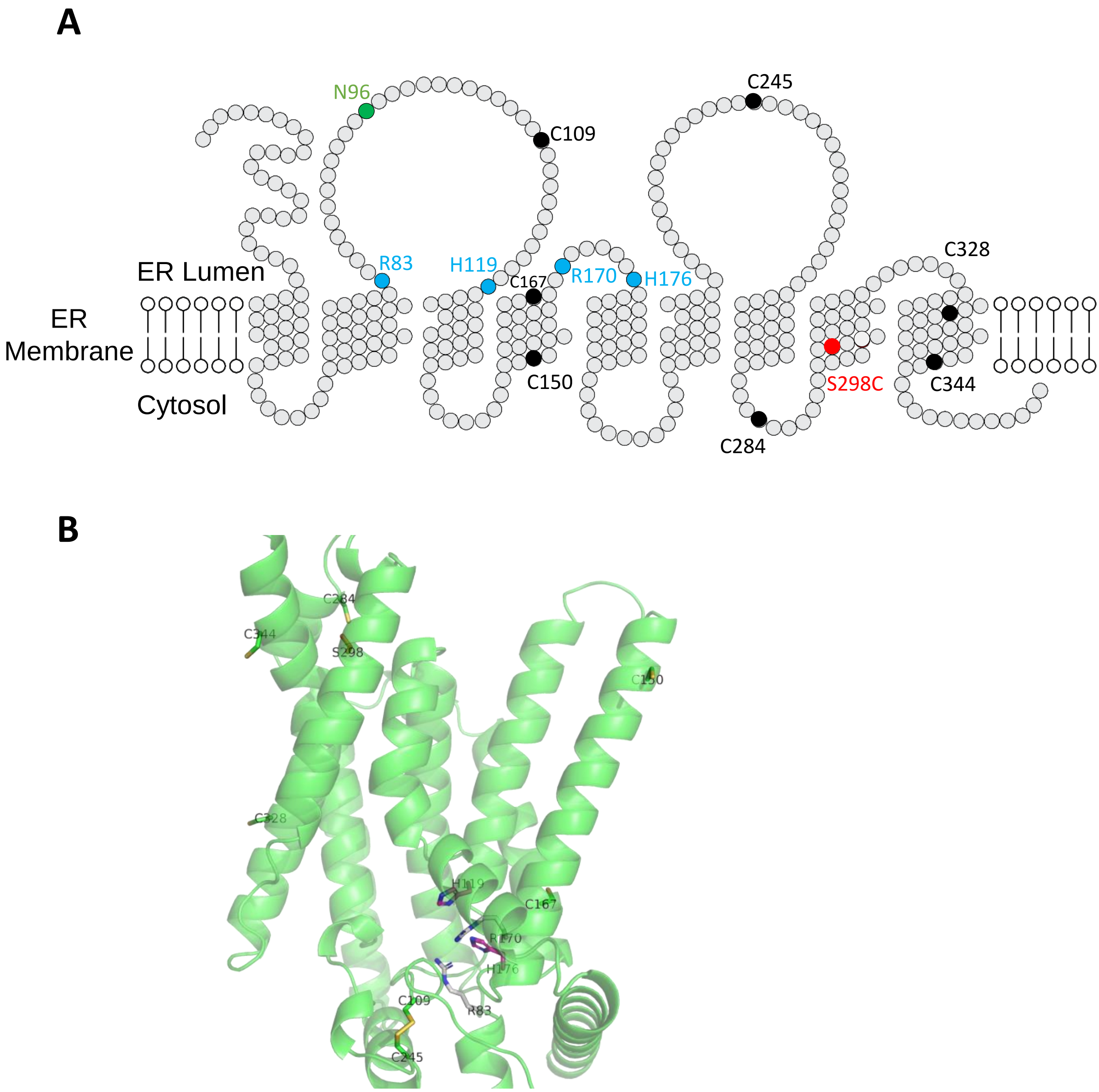

Supplementary Figure 3. Predicted topology (A) and AlphaFold structure model (B) of hG6Pase-α with annotation on locations of cysteine residues. A Topological analysis shows that hG6Pase-α is a nine-transmembrane spanning enzyme anchored in the ER membrane. Blue dots: residues directly involved in enzymatic activity. Red dot: location of S298C substitution. Black dots: location of other cysteine residues. B Predicted 3D structure model of hG6Pase-α by AlphaFold. The locations of active sites, S298, and other cysteine residues are labeled in this model. The model is available at https://alphafold.ebi.ac.uk/entry/P35575.
